# Predicting patient dropout: a nomogram for loss to follow-up after *Helicobacter pylori* eradication therapy

**DOI:** 10.3389/fpubh.2026.1736796

**Published:** 2026-02-04

**Authors:** Xiao Zhao, Xiao She, Haiyan Yang, Jing Zhao, Shi Cheng, Haitao Guan, Ping Zhao

**Affiliations:** 1 Department of Gastroenterology, The Second Affiliated Hospital of Xi’an Jiaotong University, Xi’an, Shaanxi, China; 2 Magnetic Resonance Room, Shaanxi Provincial People's Hospital, Xi’an, Shaanxi, China; 3 Gastroenterology Department II, Shaanxi Provincial People's Hospital, Xi’an, Shaanxi, China; 4 Department of Surgical Oncology, The Second Affiliated Hospital of Xi’an Jiaotong University, Xi’an, Shaanxi, China

**Keywords:** Helicobacter pylori, bismuth-containing quadruple therapy, loss to follow-up, nomogram, predictive model

## Abstract

**Background:**

*Helicobacter pylori* (*H. pylori*) infection remains a global public health burden, particularly in developing countries. While its eradication is a cornerstone for gastric cancer prevention, management is challenged by high infection rates, rising antibiotic resistance, and suboptimal treatment efficacy. Compounding these issues, patient loss to follow-up (LTFU) has emerged as a critical factor directly undermining the success of eradication therapy.

**Objective:**

This study aimed to investigate the risk factors associated with LTFU after *H. pylori* eradication, and to develop a predictive model for assessing the risk of LTFU.

**Methods:**

We conducted a prospective cohort study (April 2023-September 2024) enrolling treatment-naïve patients from a tertiary gastroenterology clinic. Following data collection via questionnaires and follow-ups, a nomogram for predicting loss to follow-up (LTFU) was developed by applying LASSO regression for variable selection and logistic regression for model building. The model was evaluated by its area under the ROC curve (AUC), calibration, and decision curve analysis (DCA), with internal validation performed via 500 bootstrap resamples to confirm reliability.

**Results:**

A total of 145 (37.76%) patients failed to follow up. From 19 potential predictors, 6 variables were independent predictive factors. They were included in the risk score: BMI > 30 kg/m^2^ (OR = 3.81, 95% CI: 1.16–12.50), government employee (OR = 2.10, 95% CI: 1.21, 3.63), distance to hospital >10 km (OR = 11.27, 95%CI: 6.29–20.18), alcohol consumption (OR = 1.82, 95% CI: 1.19–2.79), outpatient waiting time (OR = 1.01, 95% CI: 1.00–1.02), and lack of awareness of follow-up (OR = 3.32, 95% CI: 1.93–5.69). In the training set, the model demonstrated an AUC of 0.885 (95% CI: 0.843–0.918), with a sensitivity of 93.58% and a specificity of 67.92%. Comparatively, in the test set, the model achieved an AUC of 0.862 (95% CI: 0.794–0.925), with a sensitivity of 83.33% and a specificity of 77.50%, effectively forecasting the risk of patient LTFU in *H. pylori* eradication. DCA demonstrated the favorable clinical utility of the nomogram, suggesting its potential as a valuable auxiliary tool for predicting the risk of LTFU.

**Conclusion:**

The nomogram effectively assessed the risk of LTFU after *H. pylori* eradication, thereby contributing to improved treatment management outcomes.

## 1 Introduction

*Helicobacter pylori (H. pylori)* infection is closely associated with the pathogenesis of various gastrointestinal conditions, including chronic gastritis, peptic ulcers, gastric cancer, and a range of extra-gastric diseases (1, 2). Due to its established carcinogenic potential, the World Health Organization (WHO) has classified *H. pylori* as a Group I carcinogen (3). Globally, the prevalence of *H. pylori* infection has declined, decreasing from 58.20% during 1980–1990 to 43.10% in the period 2011–2022 (4). Despite this overall decrease, significant regional disparities persist. Infection rates remain substantially higher in developing countries compared to developed nations. In China, the epidemiological pattern of *H. pylori* infection exhibits a distinct north–south gradient, with generally higher prevalence observed in northern regions. Recent studies report infection rates ranging from 30.53 to 60.8% across different parts of the country (5–8). This substantial and uneven disease burden underscores the urgent need for scientifically informed public health strategies to mitigate the impact of *H. pylori* infection.

*Helicobacter pylori* infection initiates a pathological cascade in the gastric mucosa, progressing from chronic active gastritis to atrophic gastritis, intestinal metaplasia, and dysplasia, which may ultimately lead to gastric carcinogenesis (9). Eradication therapy is crucial in mitigating this progression by reducing gastric mucosal inflammation, accelerating ulcer healing, and reducing the recurrence of peptic ulcers and associated complications such as bleeding or perforation. Furthermore, such treatment can inhibit, or even partially reverse, the development of mucosal atrophy and intestinal metaplasia, thereby contributing to a reduction in the incidence of gastric cancer and related mortality. Despite these established benefits, *H. pylori* eradication efforts in China encounter significant challenges. The persistent challenges of high infection rates, heightened pathogenicity of prevalent strains, and suboptimal treatment efficacy collectively continue to pose a formidable public health threat. A major contributing factor is the increasing prevalence of antibiotic resistance, particularly to clarithromycin, levofloxacin, and metronidazole, which severely compromises the efficacy of standard therapeutic regimens.

There is some evidence reported that (10, 11) indicates a concerning rate of LTFU after initial *H. pylori* eradication, ranging from 14.30 to 32.10%. This high attrition rate poses a significant obstacle to effective long-term infection control. Failure to ensure follow-up delays necessary medical intervention for infected individuals, facilitates ongoing community transmission, and elevates the risk of antibiotic resistance development. Subsequent retreatment in patients initially lost to follow-up is often less effective, likely due to selection for resistant strains during periods of unmonitored infection or after incomplete treatment cycles. Current understanding of post-treatment attrition is primarily derived from studies on chronic infectious diseases such as tuberculosis and HIV. Established risk factors in these contexts encompass a range of demographic, behavioral, and socioeconomic variables, including male sex, extreme age groups, tobacco use, alcohol consumption, lower socioeconomic status, malnutrition, adverse drug effects, and geographical barriers to healthcare access (12–17).

However, there is currently a lack of published data on the incidence and predictors of LTFU after *H. pylori* eradication therapy. Thus, we conducted a study to investigate the rate and risk factors associated with post-treatment attrition among individuals with *H. pylori* infection in Xi’an, China. Furthermore, we developed a nomogram to predict the probability of LTFU, aiming to facilitate early identification of high-risk patients and guide targeted interventions to improve follow-up rates.

## 2 Methods

### 2.1 Study design and populations

A prospective single-center cohort study was conducted at the Gastroenterology outpatient clinic of the Second Affiliated Hospital of Xi’an Jiaotong University. Between April 2023 and September 2024, 425 patients with *H. pylori* infection, who were initiating their first course of eradication therapy, were consecutively enrolled.

#### 2.1.1 Inclusion criteria

(1) Undergoing their first course of *H. pylori* eradication therapy; (2) Prescribed an empirical bismuth-containing quadruple regimen (amoxicillin + clarithromycin + ilaprazole + colloidal bismuth pectin); (3) Capable of understanding the questionnaire content and providing accurate responses; (4) Provided written informed consent for the eradication therapy and agreed to participate in both outpatient and telephone follow-up surveys.

#### 2.1.2 Exclusion criteria

(1) Missing or erroneous baseline information, unreachable by phone, or refusal to participate in the follow-up survey after contact; (2) Withdrawal during the study due to severe organ dysfunction, allergic drug reactions, disease progression, or other serious adverse events necessitating discontinuation of the protocol therapy; (3) Presence of cognitive impairment, severe hepatic or renal disease, or other significant chronic comorbidities; (4) Planning for pregnancy, current pregnancy, or lactation; and (5) History of gastrectomy.

### 2.2 Definitions

In this study, LTFU was defined as failure to complete the recommended confirmatory test within 8 weeks after completing the initial eradication therapy, or adherence to less than 80% of the prescribed medication. This definition is adapted from established criteria in the field to ensure accurate assessment of initial treatment failure versus later reinfection (11, 18–21).

Occupation was classified into a binary variable: government employee VS. Non-government employee (which encompassed all other occupations, including self-employed, corporate workers, farmers, etc.). This was done to test the potential influence of socioeconomic and occupational factors on follow-up adherence.

Payment method was categorized as “Out-of-pocket” or “Medical insurance” to evaluate the potential influence of financial burden on follow-up adherence.

Time to hospital was defined as the patient’s self-reported one-way travel time from their residence to the hospital under normal traffic conditions, measured in minutes. This measure reflects the actual door-to-door duration, which may include in-transit time and potential waiting periods, rather than mere geographical distance.

### 2.3 Ethical considerations

This prospective cohort study was conducted in accordance with the principles of the Declaration of Helsinki and received approval from the Medical Ethics Committee of the Second Affiliated Hospital of Xi’an Jiaotong University (Approval No. 2023231). The study protocol was prospectively registered with the Chinese Clinical Trial Registry (Registration number: ChiCTR2500096567). We received informed consent from all participants.

### 2.4 Data collection procedure

All participants completed a structured questionnaire under the guidance of uniformly trained research staff. The questionnaire was developed based on the survey instrument used in the National Epidemiological Investigation of *H. pylori* Infection in China. Data were collected through one-on-one interviews, and questionnaires were reviewed on-site upon completion to ensure completeness. In cases of missing or unclear information, follow-up contacts were made to supplement the data; otherwise, incomplete questionnaires were excluded from the analysis.

### 2.5 Data management and statistical analysis

The data were entered and verified using EpiData 3.2 software. All statistical analyses were performed via the R statistical package and EmpowerStats 4.2 software. Continuous variables were expressed as mean ± SDs and compared using the Student’s *t*-test or Mann–Whitney U test, as appropriate. Categorical variables were presented as frequencies and percentages and compared using the chi-square test or Fisher’s exact test.

The Least Absolute Shrinkage and Selection Operator (LASSO) regression method was applied to screen potential predictors. Variables with non-zero coefficients in the LASSO regression were subsequently entered into a multivariate logistic regression model to construct the final prediction model (22). The model’s discriminative ability was evaluated using the area under the receiver operating characteristic curve (AUC), with internal validation performed via the bootstrap method (500 resamples). Calibration curves and a decision curve analysis (DCA) were used to measure the predictive performance of the nomogram. Clinical utility was assessed using decision curve analysis (DCA). *p* < 0.05 was considered statistically significant.

## 3 Results

### 3.1 Demographic and clinical characteristics

**Figure 1:**
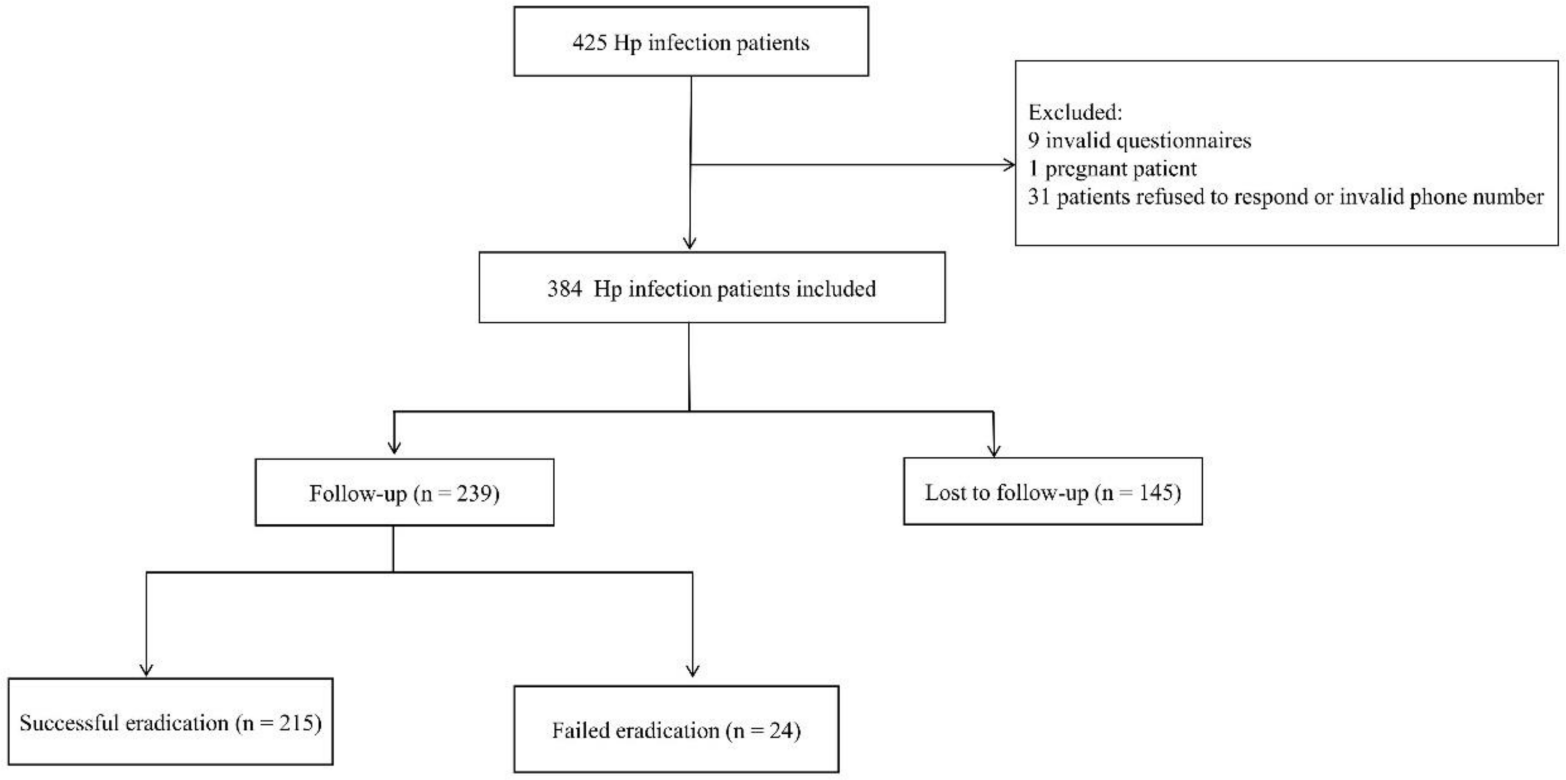
A flow chart shows the inclusion and exclusion criteria.

The flow chart of patients throughout this analysis is reported in Figure 1. The demographic and clinical characteristics of the participants are presented in Table 1. Among the 425 patients initially enrolled, 41 were excluded for the following reasons: 9 patients provided invalid questionnaires, 1 patient was excluded due to pregnancy, and 31 patients could not be contacted (refused to respond or provided invalid phone numbers). The final analytical cohort therefore consisted of 384 participants, who were randomly allocated into a training group (*n* = 268) and a test group (*n* = 116). In the training and test groups, 40.67% (109/268) and 31.03% (36/116) of participants were LTFU, respectively. Common reasons for attrition included being too busy at work, failure to initiate medication, and resolution or absence of symptoms. After comparing the baseline data of both groups, it was found that there were no significant differences in sex, age, BMI, type of work, residence, payment method, occupation, education, marital status, monthly household income per capita, smoking status, alcohol consumption, distance to the hospital, outpatient waiting time, time to hospital, and various medical histories (prior *H. pylori* infection, family history of *H. pylori*, family history of gastric disease), indicating a generally comparable outcome distribution between the two sets for model building.

**Table 1:** Demographic and clinical characteristics of patients.

Variables	Training group (*n* = 268)	Test group (*n* = 116)	*p*
Age, y	39.64 ± 11.64	38.78 ± 11.25	0.505
Sex			0.690
Male	133 (49.63%)	55 (47.41%)	
Female	135 (50.37%)	61 (52.59%)	
BMI, Kg/m2			0.983
≤30	254 (94.78%)	110 (94.83%)	
>30	14 (5.22%)	6 (5.17%)	
Type of work			0.132
Mental labor	198 (73.88%)	94 (81.03%)	
Physical labor	70 (26.12%)	22 (18.97%)	
Residence			0.077
Urban	239 (89.18%)	110 (94.83%)	
Rural	29 (10.82%)	6 (5.17%)	
Payment method			0.142
Medical insurance	147 (54.85%)	73 (62.93%)	
Out-of-pocket	121 (45.15%)	43 (37.07%)	
Occupation			0.688
Non-government employee	191 (71.27%)	85 (73.28%)	
Government employee	77 (28.73%)	31 (26.72%)	
Education			0.697
High school/technical secondary school or below	65 (24.25%)	26 (22.41%)	
College or higher education	203 (75.75%)	90 (77.59%)	
Marital status			0.782
Married	188 (70.15%)	83 (71.55%)	
Unmarried/Divorced or separated/Widowed	80 (29.85%)	33 (28.45%)	
Monthly household income per capita (CNY)			0.545
<2000	29 (10.82%)	9 (7.76%)	
2000–5,000	85 (31.72%)	38 (32.76%)	
5,000–1,000	107 (39.93%)	53 (45.69%)	
>10,000	47 (17.54%)	16 (13.79%)	
Smoking status			0.178
No	221 (82.46%)	102 (87.93%)	
Yes	47 (17.54%)	14 (12.07%)	
Alcohol consumption			0.870
No	171 (63.81%)	73 (62.93%)	
Yes	97 (36.19%)	43 (37.07%)	
Distance to hospital (km)			0.440
≤10	148 (55.22%)	69 (59.48%)	
>10	120 (44.78%)	47 (40.52%)	
Outpatient waiting time (min)	25.36 ± 28.29	27.13 ± 24.01	0.092
Time to hospital (minutes)	60.92 ± 67.39	55.35 ± 63.47	0.887
Prior *H. pylori* infection			0.738
No	178 (66.42%)	75 (64.66%)	
Yes	90 (33.58%)	41 (35.34%)	
Family history of *H. pylori* infection			0.305
No	197 (73.51%)	91 (78.4:5:%)	
Yes	71 (26.49%)	25 (21.55%)	
Family history of gastric disease			0.576
No	208 (77.61%)	93 (80.17%)	
Yes	60 (22.39%)	23 (19.83%)	
Awareness of follow-up			0.784
Important	184 (68.66%)	78 (67.24%)	
Unimportant	84 (31.34%)	38 (32.76%)	
LTFU			0.:074
No	159 (59.33%)	80 (68.97%)	
Yes	109 (40.67%)	36 (31.03%)	

### 3.2 Predictor selection and model construction

**Figure 2:**
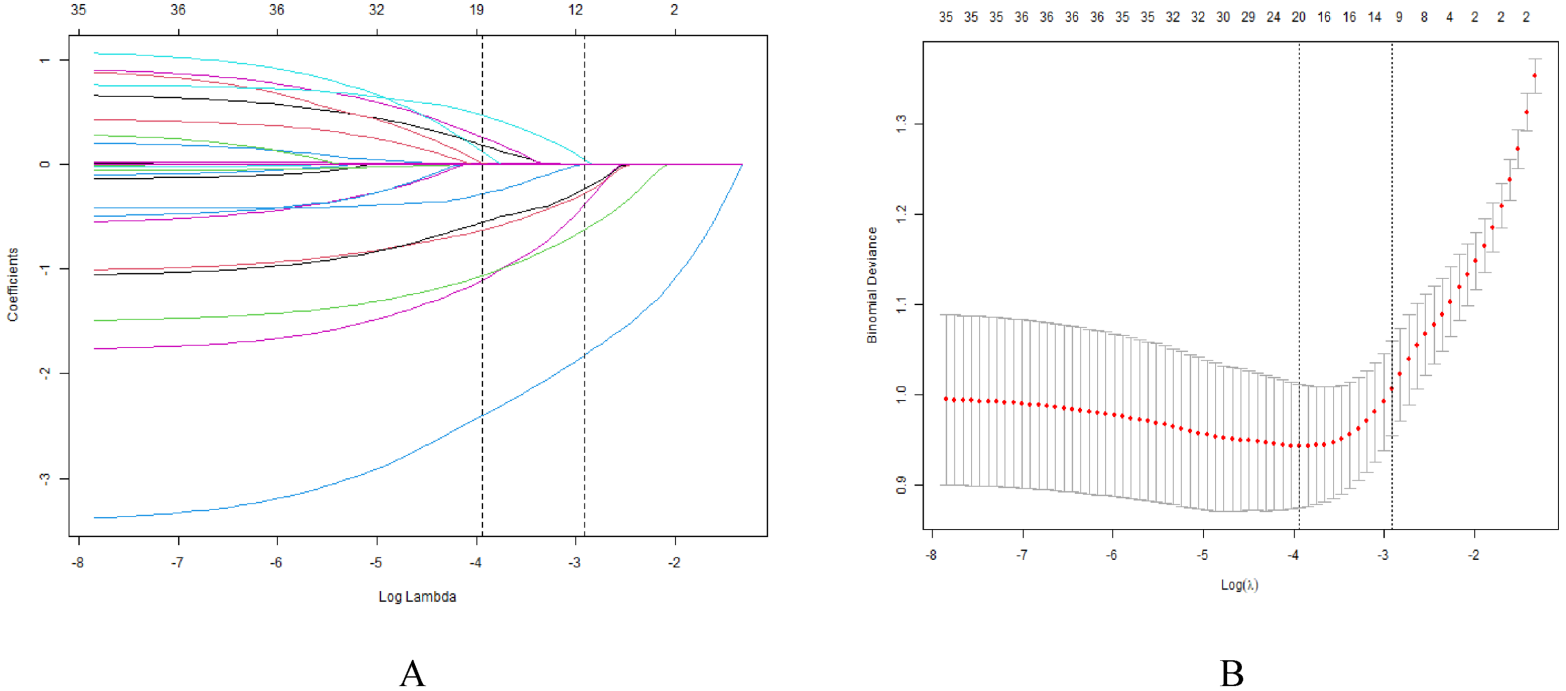
The LASSO regression analysis identified variables predicting LTFU. **(A)** Number of non-zero coefficients in the model. **(B)** Number of variables corresponding to different *λ* values. Seven variables were selected by LASSO regression and constituted the basic factors of the prediction model.

To minimize the risk of overfitting, a LASSO regression analysis was employed. This approach identified seven robust predictors of LUFU from the 19 baseline variables (Table 1; Figure 2): BMI, occupation, distance to the hospital, outpatient waiting time, alcohol consumption, type of residence, and awareness of the need for follow-up.

**Figure 3:**
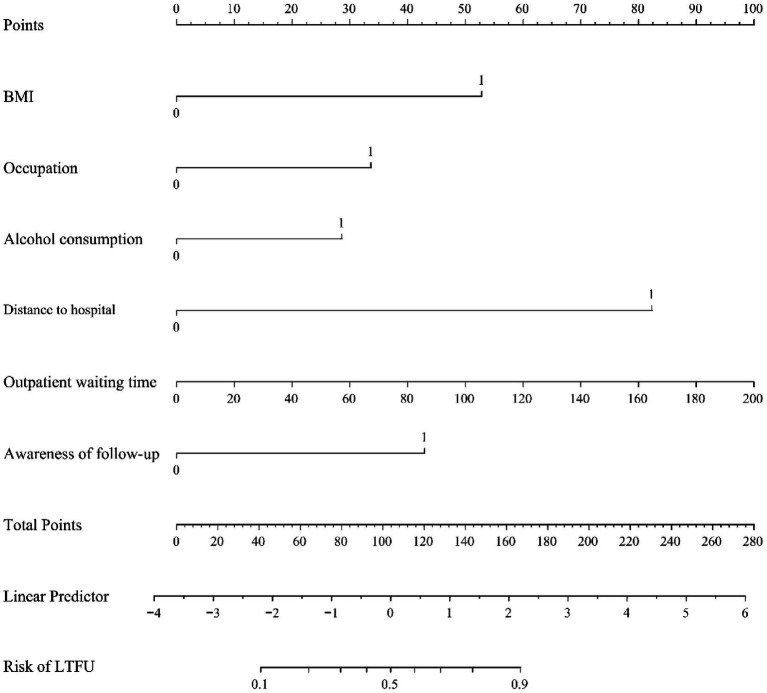
Nomogram for predicting LTFU in patients with *H. pylori* eradication. Instructions: Factors include BMI, occupation, distance to hospital, outpatient waiting time, alcohol consumption, and awareness of follow-up—points assigned by that variable value. Then, the points from each variable value were summed. The sum on the total points scale was located and vertically projected onto the bottom axis, and then a personalized risk for LTFU was obtained.

After multivariate analysis, the independently statistically significant predictors of LTFU were BMI, occupation, distance to hospital, outpatient waiting time, alcohol consumption, and awareness of follow-up (Table 2). The final model is expressed as: logit (LTFU) = −7.29047 + 1.84325*BMI + 1.17386*occupation +0.99756*alcohol consumption +2.87117*distance to hospital +0.01744*outpatient waiting time +1.49824*awareness of follow-up (Figure 3).

**Table 2:** Prediction factors for LTFU after *H. pylori* eradication.

Variables	OR (95% CI)	*p*
BMI, Kg/m^2^		
≤30	1.0	
>30	3.81 (1.16, 12.50)	0.0276
Residence		
Urban	1.0	
Rural	0.55 (0.23, 1.30)	0.1747
Occupation		
Non-government employee	1.0	
Government employee	2.10 (1.21, 3.63)	0.0083
Alcohol consumption		
No	1.0	
Yes	2.27 (1.36, 3.79)	0.0017
Distance to hospital (km)		
≤10	1.0	
>10	11.27 (6.29, 20.18)	<0.0001
Outpatient waiting time (min)	1.01 (1.00, 1.02)	0.0035
Awareness of follow-up		
Important	1.0	
Unimportant	3.32 (1.93, 5.69)	<0.0001

Adjust model for: age.

### 3.3 The performance of the LTFU nomogram

**Figure 4:**
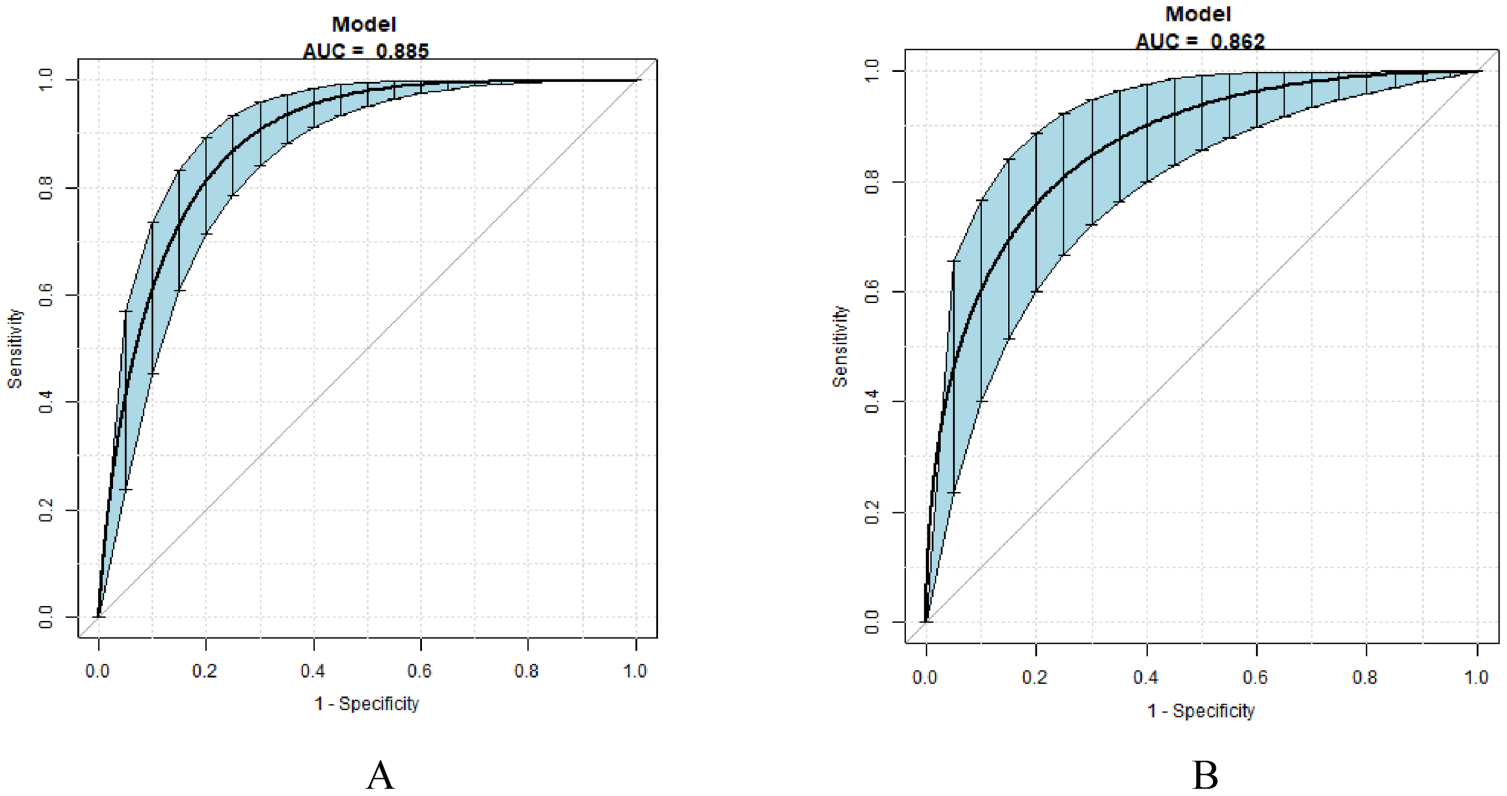
The ROC curve after internal validation using bootstrap resampling (times = 500). **(A)** ROC curves of the nomogram in the training group. **(B)** ROC curve of the nomogram in the test group.

**Figure 5:**
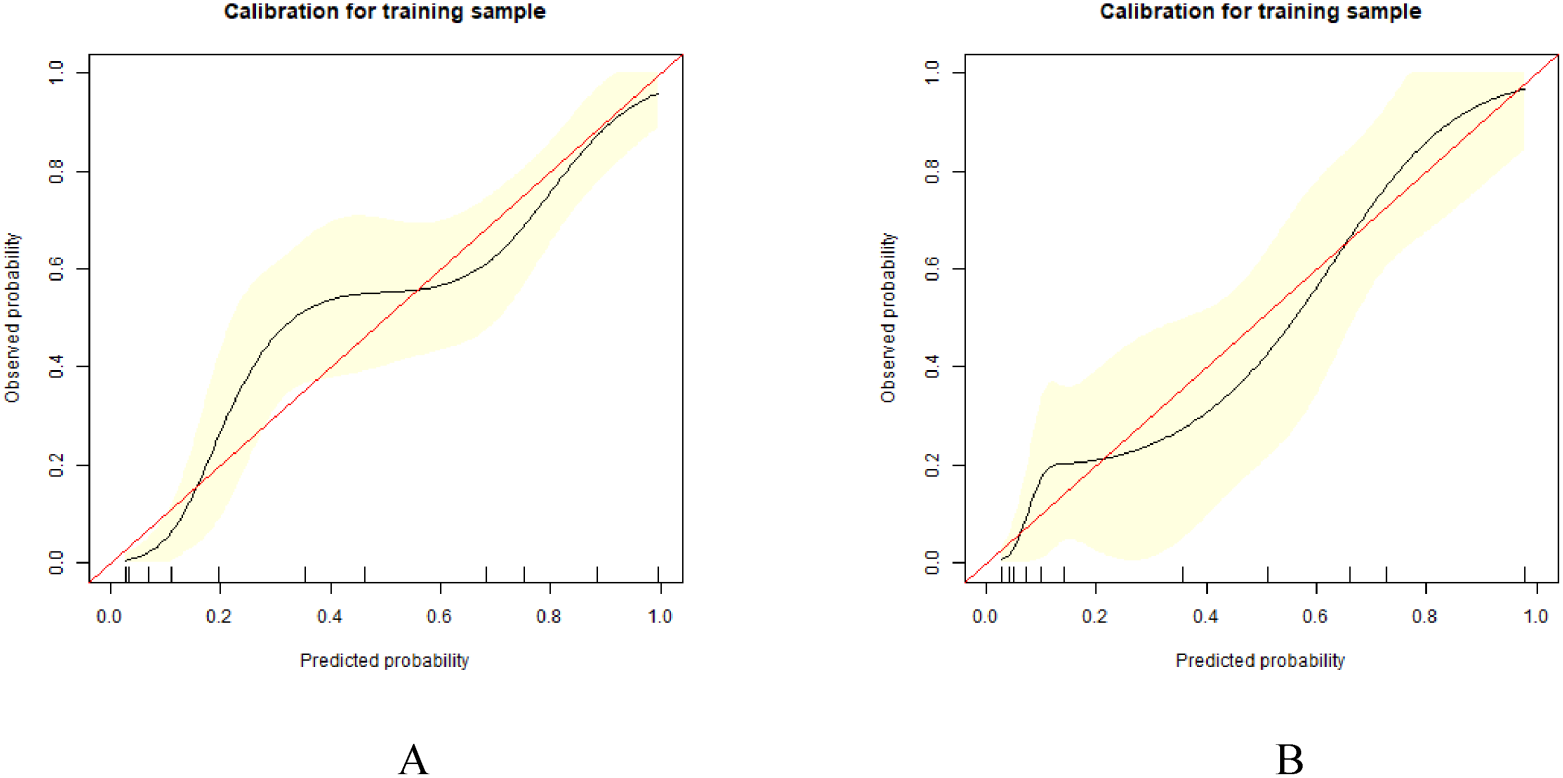
Calibration curves of the nomogram. **(A)** Calibration curve of the predictive model in the training group. **(B)** Calibration curve of the predictive model in the test group.

The prediction model is verified internally. Internal verification is done through bootstrapping, times = 500. In the training set, the model demonstrated an AUC of 0.885 (95% CI: 0.843–0.918), with a sensitivity of 93.58% and a specificity of 67.92%. Comparatively, in the test set, the model achieved an AUC of 0.862 (95% CI: 0.794–0.925), with a sensitivity of 83.33% and a specificity of 77.50% (Figure 4). The calibration curves showed that the predicted probabilities were closely aligned with the observed outcomes across a range of predicted probabilities, confirming the model’s accuracy in estimating LTFU risk (Figure 5). Effectively forecasting the risk of patient LTFU in *H. pylori* eradication.

**Figure 6:**
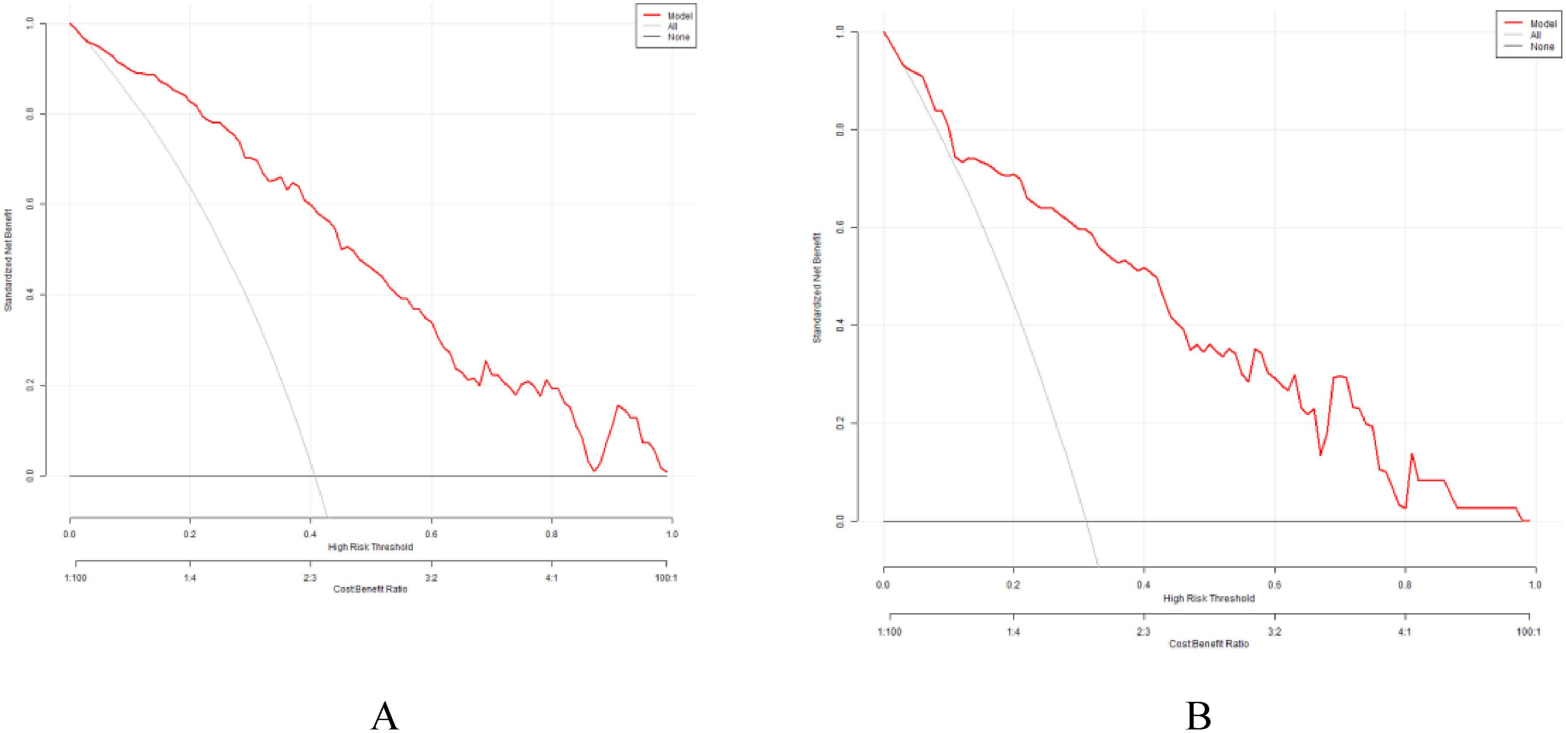
Decision curve of the nomogram. **(A)** Decision curves of the nomogram in the training group. **(B)** Decision curves of the nomogram in the test group.

To evaluate clinical utility, DCA was performed on both the training and validation cohorts. As shown in Figure 6, the DCA results demonstrated that the proposed model yielded favorable clinical utility in both the training and validation cohorts, providing meaningful support for individualized risk assessment and clinical decision-making across reasonable threshold ranges.

## 4 Discussion

This study firstly addresses the critical clinical challenge of follow-up adherence after *H. pylori* eradication by developing the nomogram to predict the risk of LTFU. Based on six readily available variables, the model demonstrated robust discriminative ability (AUC: 0.885 training, 0.862 validation), good calibration, and meaningful clinical utility via decision curve analysis. This tool provides a quantitative method for the early identification of high-risk individuals in resource-constrained settings. By enabling risk-stratified management, it facilitates targeted interventions—such as prioritized reminders or enhanced patient education—thereby optimizing the allocation of clinical resources, supporting sustained patient engagement, and ultimately aiming to improve follow-up rates and patient outcomes.

### 4.1 Interpretation of key predictors and clinical context

Consistent with prior evidence (23, 24), our study found a travel distance of >10 km to the healthcare facility as an independent risk factor for LTFU. Patients residing farther away face greater time commitments and financial costs for follow-up visits, which may further reduce adherence to post-eradication assessments. Furthermore, Denis Opio et al. (25) found that long outpatient waiting time was a risk factor for LTFU, which is consistent with our study.

Alcohol consumption is a known risk factor for *H. pylori* management (26, 27). In our study, alcohol use significantly increased the likelihood of LTFU, with drinkers exhibiting a 1.82-fold higher risk than non-drinkers. This is consistent with previous research (28, 29). One possible explanation is that alcohol use often co-occurs with poor mental health, including anxiety (30) and depression (31), which may diminish a patient’s motivation to attend follow-up appointments.

Obesity is reported to increase the incidence of LTFU, Torrens C. et al. (32) found severe obesity (BMI > 35 kg/m^2^) was associated with a 2.44-fold increase in LTFU risk. Vejrup K. et al. (33) also found that individuals with higher BMI were more likely to be LTFU. These findings are in line with our results. Obesity is closely linked to metabolic syndrome (34), and patients with obesity often carry a higher burden of comorbidities, which may further affect their ability or willingness to adhere to follow-up schedules. However, contrasting results have been reported by Soedarsono et al. (35) and Frijtersa et al. (36), who found that underweight BMI was associated with LTFU. Thus, the relationship between BMI and LTFU warrants further investigation.

Interestingly, we observed unexpectedly that civil servants were more likely to be lost to follow-up compared with other occupational groups. Similarly, consistent with Mulat A et al. (37), who also reported higher LTFU among government employees. This may reflect competing demands and institutional constraints rather than lack of access: despite having health coverage and formal leave, rigid work schedules and high occupational demands may impede attendance for non-acute appointments. Additionally, reliance on annual employer-organized health checks might lead to underestimation of the need for timely, condition-specific follow-up. In contrast, Soedarsono et al. (35) and Frijters et al. (36) found that unemployment or informal employment was associated with LTFU. Therefore, the influence of occupation on LTFU remains complex and merits further research.

### 4.2 Clinical utility, implementation, and strategies for high-risk patients

The primary value of this nomogram lies in enabling risk-stratified patient management. By providing a quantitative, individualized risk score at the point of prescription, it shifts the clinical approach from a uniform, reactive follow-up protocol to a proactive, resource-efficient strategy. For patients stratified as high-risk, targeted interventions can be immediately deployed: (1) Enhanced Pre-treatment Counseling: Structured education emphasizing the critical importance and specific timing of the confirmatory test; (2) Proactive Engagement: Implementing scheduled check-in calls or messages during the treatment course to address side effects and reinforce adherence. (3) Reducing Logistical Barriers: Exploring flexible scheduling, telehealth options for post-therapy consultation, or facilitating testing at more convenient locations. (4) Digital Reinforcement: Automated SMS or application-based reminders tailored to high-risk individuals.

This approach optimizes the allocation of limited clinical resources (e.g., nursing time, digital health infrastructure) to the patients who need them most, aiming to improve follow-up completion rates and, consequently, long-term clinical outcomes.

### 4.3 Methodological considerations: definition of LTFU

In defining LTFU as a missed confirmatory test within 8 weeks post-therapy, we aimed to balance clinical guidelines with diagnostic accuracy. This operational definition, adapted from established criteria (11), is designed to maximize the likelihood that a positive test result within this window reflects true eradication failure rather than early reinfection, a distinction that becomes less clear beyond this period (21). While this timeframe is clinically reasoned for our setting, its optimality may vary in different healthcare contexts and remains a parameter for consideration in future research or implementation.

### 4.4 Strengths and limitations

Key strengths of this study include its prospective design, the incorporation of diverse and clinically relevant predictors, rigorous internal validation using bootstrapping, and evaluation of clinical utility via decision curve analysis. The nomogram offers an intuitive, visual tool that could be integrated into routine practice. The study has several limitations. Its prospective cohort design is inherently resource-intensive. Moreover, the reliance on questionnaire-based data collection led to the exclusion of participants with incomplete responses, potentially introducing selection bias. Most importantly, the predictive nomogram was developed and internally validated within a single-center context. Consequently, its generalizability to other healthcare settings remains unestablished and warrants external validation through future multi-center studies.

## 5 Conclusion

In conclusion, BMI, occupation, distance to hospital, outpatient waiting time, alcohol consumption, and awareness of follow-up were identified as predictive factors of LTFU. Based on these factors, we constructed a robust and clinically applicable nomogram that accurately estimates the individual risk of LTFU, thus offering potential for targeted intervention in clinical practice.

## Data Availability

The data used to support the findings of this study can be available from the corresponding author upon reasonable request. Requests to access the datasets should be directed to Ping Zhao, peggyzhao@163.com.
